# Adenomyosis and endometriosis. Re-visiting their association and further insights into the mechanisms of auto-traumatisation. An MRI study

**DOI:** 10.1007/s00404-014-3437-8

**Published:** 2014-09-21

**Authors:** G. Leyendecker, A. Bilgicyildirim, M. Inacker, T. Stalf, P. Huppert, G. Mall, B. Böttcher, L. Wildt

**Affiliations:** 1 Kinderwunschzentrum (Fertility Center) Darmstadt, Bratustr. 9, 64293 Darmstadt, Germany; 2 Institute of Diagnostic and Interventional Radiology, Academic Teaching Hospital to the Universities of Frankfurt/Main and Heidelberg/Mannheim, Grafenstr. 7, 64283 Darmstadt, Germany; 3 Institute of Pathology, Klinikum Darmstadt, Academic Teaching Hospital to the Universities of Frankfurt/Main and Heidelberg/Mannheim, Grafenstr. 7, 64283 Darmstadt, Germany; 4 Department of Obstetrics and Gynecology, University Clinic of Gynecological Endocrinology and Reproductive Medicine, Medical University Innsbruck, Anichstrasse 35, 6020 Innsbruck, Austria

**Keywords:** Adenomyosis, Endometriosis, Auto-traumatization, Dysmenorrhea, Pathophysiology, MRI

## Abstract

**Purpose:**

In a series of publications, we had developed the concept that uterine adenomyosis and pelvic endometriosis as well as endometriotic lesions at distant sites of the body share a common pathophysiology with endometriosis constituting a secondary phenomenon. Uterine auto-traumatization and the initiation of the mechanism of tissue injury and repair (TIAR) were considered the primary events in the disease process. The present MRI study was undertaken (1) to corroborate this concept by re-visiting, in view of discrepant results in the literature, the association of adenomyosis with endometriosis and (2) to extend our views concerning the mechanisms of uterine auto-traumatization.

**Patients and methods:**

MRI was performed in 143 women attending our center, in whom, on the basis of transvaginal sonography (TVS) and historical data, such as documented endometriosis and dysmenorrhea of various degrees of severity, the presence of uterine adenomyosis was suspected. In addition to the measurement of the diameter of junctional zone (JZ) of the anterior and posterior walls in the mid-sagittal plane, the diagnosis of adenomyosis was based on visualization, in that all planes were analyzed with scrutiny. By this method of “visualization” all transient enlargement of the JZ, such as peristaltic waves of the archimyometrium and sporadic neometral contractions that might mimic adenomyotic lesions could be excluded. At the same time, this method allowed to lower the limit of detection in terms of thickness of the JZ for assured diagnosis of adenomyosis. Furthermore, the localizations of the individual lesions, their shapes and patterns were described.

**Results:**

With the method of ‘visualization’, the diagnosis of uterine adenomyosis could be verified in 127 of the 143 patients studied. The prevalence of endometriosis in adenomyosis was 80.6 % and the prevalence of adenomyosis in endometriosis was 91.1 %. As concluded from their localization within the uterine wall, the adenomyotic lesions predominantly developed in the median region of the upper two-thirds of the uterine wall. Cystic cornual angle adenomyosis was a distinct phenomenon that was only observed in patients suffering from extreme primary dysmenorrhea. Aside from this, the majority of the patients complained of primary dysmenorrhea (80 %). On the basis of these findings and the fact that particularly extreme primary dysmenorrhea is associated with high intrauterine pressure, menstrual ‘archimetral compression by neometral contraction’ has to be considered as an important cause of uterine auto-traumatization in addition to uterine peristalsis and hyperperistalsis. Both mechanical functions of the non-pregnant uterus exert their strongest power in the upper region of the uterus, which is compatible with the predominant localization of the adenomyotic lesions.

**Conclusions:**

The data confirm our previous results of a high association of adenomyosis with endometriosis and vice versa. Our view of the mechanism of uterine auto-traumatization by mechanical functions of the non-pregnant uterus has to be extended, in that ‘archimetral compression by neometral contractions’ could be realized as the predominant cause of mechanical strain to the non-pregnant uterus. The data of this study confirm our concept of the etiology and pathophysiology of adenomyosis and endometriosis in that the process of chronic proliferation and inflammation is induced at the level of the archimetra by chronic uterine auto-traumatization. Furthermore, with respect to the diagnosis of uterine adenomyosis (and consequently endometriosis) this study shows a high degree of accordance between the findings in real-time TVS and MRI.

## Introduction

In spite of continuous efforts, the etiology and pathophysiology of adenomyosis and endometriosis and their interrelationship are not fully understood. There is increasing evidence that adenomyosis constitutes an important factor of infertility. Therefore, the elucidation of its pathophysiology is of utmost clinical importance. In a series of reports, we had developed the view that these lesions are highly associated and are the results of uterine auto-traumatization [1–6]. The pathophysiological process was termed “tissue injury and repair” (TIAR) [7–9]. The present study was conducted to corroborate and extend this view.

The work of Rokitansky, von Recklinghausen, Freund and Cullen [10–16] laid the basis of the present understanding of adenomyosis as a benign uterine tumor derived from Müllerian duct tissue [17]. The gross and microscopic morphology, the expansion beyond the uterine confines, the occurrence at distant sites in the body and the main clinical characteristics were described in detail by these authors. With his basically correct and seminal concept of tubal dissemination of endometrial tissue into the peritoneal cavity, Sampson dealt therefore only with a segmental part of the disease [18]. Furthermore, he did not appreciate uterine adenomyosis as a significant component of the pathophysiological process. While it was well established that uterine adenomyosis resulted from a focal infiltration and/or broad expansion of Müllerian tissue into the underlying myometrium with continuity of the endometrial glandular structures [14, 19], he suggested uterine adenomyosis to result from vascular transmission [20]. Although the view that uterine adenomyosis and endometriosis constitute a nosological entity was never completely abandoned [21]; with time and enforced by the introduction of laparoscopy as a diagnostic procedure, Sampson’s theory led to regard endometriosis mainly as a peritoneal disease resulting from retrograde menstruation, while uterine adenomyosis nearly fell into oblivion [8, 22–24]. Until the beginning of this century much scientific effort was laid on corroborating Sampson’s concept [25]. A re-consideration of the uterus as a significant determinant in the disease process was initiated, when it was shown that the non-pregnant uterus is not a quiescent organ, but rather actively involved in the early process of reproduction by mechanical functions that were found to be severely impaired or dysfunctional in patients with endometriosis [26–30]. Research efforts using a broad array of methods resulted in a more precise understanding of the disease process that led again to the inclusion of uterine adenomyosis into the pathophysiology of endometriosis [8]. Continuously improved imaging techniques, such as transvaginal sonography and MRI, significantly contributed to this new understanding [31–35]. Of paramount importance in gaining new insights into the pathophysiology of endometriosis and adenomyosis was the identification and detailed description of the endometrial–subendometrial unit or archimetra [36]. This evolutionarily and ontogenetically oldest part of the uterus is physiologically continuously subjected to mechanical strain during the reproductive phase of life [7–9].

In studies addressing the prevalence of adenomyosis in endometriosis and vice versa, discrepant results were obtained [5, 37]. Since the diagnosis of adenomyosis is highly dependent upon the diagnostic criteria applied, it appeared to be necessary to re-evaluate the association of adenomyosis with endometriosis. Special emphasis was laid on the signs of early uterine adenomyosis as determined by MRI.

In our previous studies, we had initially focused on uterine peristalsis and hyperperistalsis as the main causes of uterine auto-traumatization within the process of tissue injury and repair (TIAR) [6–8, 38]. In the meantime, however, it became evident that, in addition to uterine peristalsis for directed sperm transport, the contractile activity of the uterus during menstruation constitutes another important mechanical function of the non-pregnant uterus [9]. A considerable number of women experience these contractions as primary dysmenorrhea of various strengths, indicating their enormous mechanical power [39]. Thus, neometral menstrual contractions could contribute to uterine auto-traumatization and, therefore, considered as an important component of the TIAR process.

In addition, instead of merely studying the association of adenomyosis with endometriosis, we realized the necessity to extend and to refine our view of uterine adenomyosis under the aspect of the preferential localizations of the lesions within the uterine wall. In relating the predominant localizations as well as the shapes and patterns of the lesions to the sites, at which the mechanical functions of the non-pregnant uterus presumably exerts its strongest impact on the archimetra, a deeper insight into the mechanisms of uterine auto-traumatization was attempted that would further corroborate our pathophysiological concept of uterine auto-traumatization.

## Patients and methods

In a total of 143 patients aged 23–42 years (mean age 33 years) attending the Fertility Center Darmstadt, MRI scans were performed on the basis of the following historical data.

### Clinical symptoms and findings by transvaginal sonography (TVS)

#### Transvaginal sonography

In all 143 patients, transvaginal sonograms were performed and none was considered as normal. The indication for subsequent MRI on the basis of sonographic findings were:

- enlarged uterus (fibroids excluded);
- uterine asymmetry with enlarged uterine walls (fibroids excluded);
- focally destroyed, absent or expanded uterine “halo”;
- inhomogenous texture of the myometrium;
- irregular endometrial–myometrial interface;
- cystic structures considered as myometrial endometriotic cysts.

#### Endometriosis

In 72 patients aged from 22 to 42 years (mean age 32.3 years), the presence (*n* = 56, mean age 32.2 years; range from 22 to 41 years) or absence (*n* = 16; mean age 32.7 yrs; range from 25 to 42 years) of endometriosis was documented. The majority of patients presented a surgical and histological report. Only in one case the diagnosis of endometriosis was based on visualization during laparoscopy with high credibility. In two cases, MRI findings such as infiltration of the urinary bladder and ovarian endometriomas were indicative of pelvic endometriosis.

#### Dysmenorrhea

In 116 patients, the presence or absence of dysmenorrhea was documented.

Dysmenorrhea was defined as mild with no use, moderate with occasional use and severe with permanent use of analgetic medication during every menstrual period. Extreme dysmenorrhea was characterized by absence from school or work and sometimes collaptic states. Of these 116 patients, 7 had no history of dysmenorrhea at all.

Infertility was not a criterion to perform an MRI scan, because in some patients that attended our center because of a history or of symptoms suggestive of endometriosis, infertility was not proven. Moreover, in most of the infertile couples low sperm counts of the male partners were documented.

The patients were recruited mostly by the senior investigator (G.L.) and also by other members of the medical team of the fertility center. The senior investigator did see all the patients at least twice, took their history and performed all transvaginal ultrasound scans.

## Magnetic resonance imaging (MRI)

In all 143 women the uteri were examined by means of MRI using the same techniques as published before [4, 5]. Slice thickness of t2-weighted images was 3.0 mm with a gap of 0.3 mm in sagittal orientation and 3 mm with 0.6 mm gap in transversal and coronal orientation. Additionally, we acquired t2-weighted sagittal-oblique images (3.0, 0.9 mm gap) and t1-weighted transversal images (5, 1.0 mm gap). To prevent functional alterations of the image quality regularly, butylscopolamine was administered before onset of the scanning. All diameters of the junctional zone were documented by electronic callipers and expressed in millimeters and were obtained from the uteri in a mid- or paramedian sagittal, transverse as well as in a coronary plane. Maximum diameters of the anterior and posterior walls, respectively, with respect to partial volume effect were considered. All quantitative measurements or qualitative (visualized) evidence (presence or absence of adenomyosis) were obtained separately and independently by G.L. and a radiologist. In case of discrepant findings, the respective scans were re-analyzed by G.L. and P.H.

In addition to the measurements of the diameter of the junctional zone in the posterior and anterior walls of the uterus, the localizations, shapes and patterns of the lesions were documented. For this purpose all the planes (sagittal, coronary and transverse) were analyzed carefully.

An enlargement of the junctional zone of 12 mm or more was considered as strong evidence of adenomyosis. Below this thickness, additional findings had to be present to warrant the diagnosis of adenomyosis such as cystic structures within the sub-basal myometrium, focal thickening of the junctional zone that could not be related to functional alterations such as transient contractions of the neometra or cyclic peristaltic waves, and irregular and stepwise expansions of the junctional zone [33–35]. This method was termed *diagnosis by visualization* and was applied in cases with a junctional zone diameter below 12 mm. In this study the term *“visualized adenomyosis”* is used for the cases that fulfil either the criteria of visualization or a thickness of the JZ of ≥12 mm. Functional alterations that mimicked focal adenomyosis, such as peristaltic waves and transitory contractions of the neometra, were indentified in that they had disappeared in scans performed after a lapse of time.

### Statistical analysis

Student’s *t* test was performed whenever appropriate. All patients gave their written informed consent for use of their data for scientific evaluation.

## Results

**Fig. 1:**
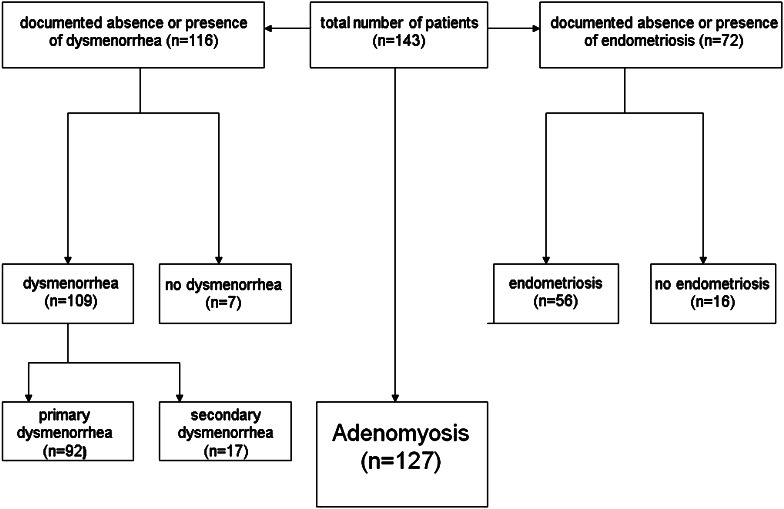
The clinical characteristics of the patients who entered the study

Figure 1 provides an overview of the study population. In 143 patients, an MRI was performed on the basis of findings on real-time vaginal sonography that were suspicious of uterine adenomyosis. In a large proportion of the patients, historical data such as documented dysmenorrhea and endometriosis were available.

### Dysmenorrhea

In 107 out of 127 patients with “visualized” adenomyosis, absence or presence of dysmenorrhea could be documented. Primary dysmenorrhea (*n* = 84; 78.5 %) was predominant over secondary dysmenorrhea (*n* = 14; 14.7 %) and within both groups severe dysmenorrhea prevailed. The extreme form (*n* = 23; 21.5 %) was only documented in women with primary dysmenorrhea. Only seven patients (6 %) were completely free of menstrual pain (Table 1).

**Table 1:** In 107 of 127 patients with “visualized” adenomyosis, the presence or absence of dysmenorrhea was documented (figures in brackets and bold)

	*n*	%
Primary dysmenorrhea		
Mild	10 (**10**)	8.6 (**9.3**)
Moderate	20 (**17**)	17.2 (**15.9**)
Severe	38 (**34**)	32.8 (**31.7**)
Extreme	24 (**23**)	20.7 (**21.5**)
Total	92 (**84**)	79.3 (**78.5**)
Secondary dysmenorrhea		
Mild	3 (**2**)	2.6 (**1.8**)
Moderate	5 (**5**)	4.3 (**4.7**)
Severe	9 (**9**)	7.9 (**8.4**)
Total	17 (**16**)	14.6 (**14.9**)
None dysmenorrhea		
None	7 (**7**)	6.0 (**6.6**)

Eighty-four patients (78.5 %) suffered from primary and 16 (14.9 %) from secondary dysmenorrhea. Seven patients reported the absence of dysmenorrhea (6.6 %). The severity of dysmenorrhea was defined according to the use of analgesics—mild: none; moderate: occasional; severe: regular; extreme: regular and absence from school and work, respectively

### Endometriosis

In 72 of the 143 patients, the absence or presence of endometriosis was documented. In 17 patients no endometriosis was found by laparoscopy. In 56 patients, endometriosis was documented (Fig. 1). In this study, unlike in a previous study [5], the stages of the disease were not specifically documented. In the meantime, we had come to the conclusion that the fragments of basal endometrium, following their transtubal dissemination, might have their own fate.

### Characterization of adenomyosis

In the study group (*n* = 143), the diameters of the junctional zone increased with age and showed a trend of being larger in the posterior than in the anterior wall (Table 2).

**Table 2:** The diameters of the junctional zone in 143 patients with suspected adenomyosis as summarized in three groups of age

Age	AW (±SD)	PW (±SD)	*n*
<30 years	9.20 (±3.21)	11.42 (±4.87)	33
30–35 years	11.31 (±5.87)	11.71 (±5.66)	68
>35 years	12.43 (±7.39)	14.46 (±7.95)	42

There is an increase in the JZ with age and a trend of the preponderance of an enlarged JZ in the posterior wall of the uterus. The differences were not significant due to the large standard deviation (SD) in each group. Prevalence of endometriosis in adenomyosis

**Fig. 2:**
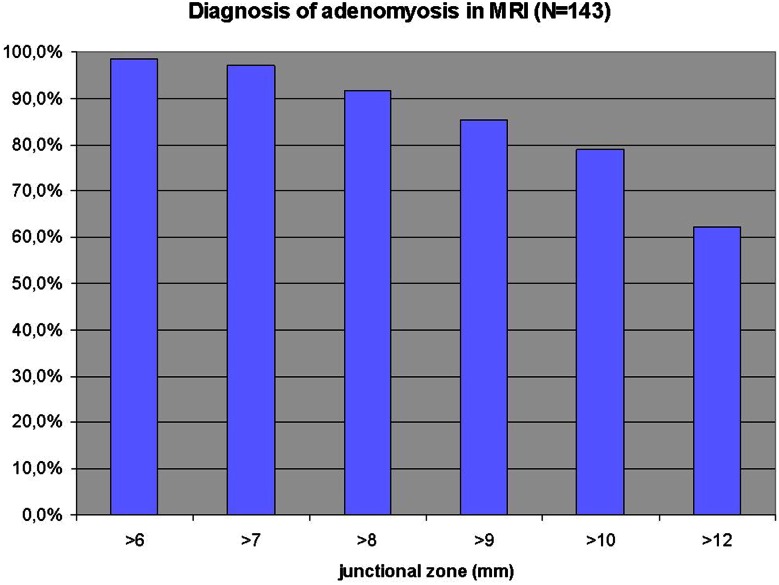
Percentage of patients with the diagnosis of adenomyosis using limits of detection based on the thickness of the junctional zone ranging from ≥6 to ≥12 mm in the sagittal plane of the anterior or posterior wall of the uterus in 143 patients

In 98 % of the 143 patients, the diameter of the junctional zone was larger than 6 mm. This percentage declined gradually with the stepwise increase in millimeters of the arbitrary detection limit and fell to 62 % when the diagnosis of adenomyosis was defined to be established at a thickness of the junctional zone of at least 12 mm. In 30 % of the patients, the diameter of the junctional zone was between 8 and 12 mm (Fig. 2).

**Fig. 3:**
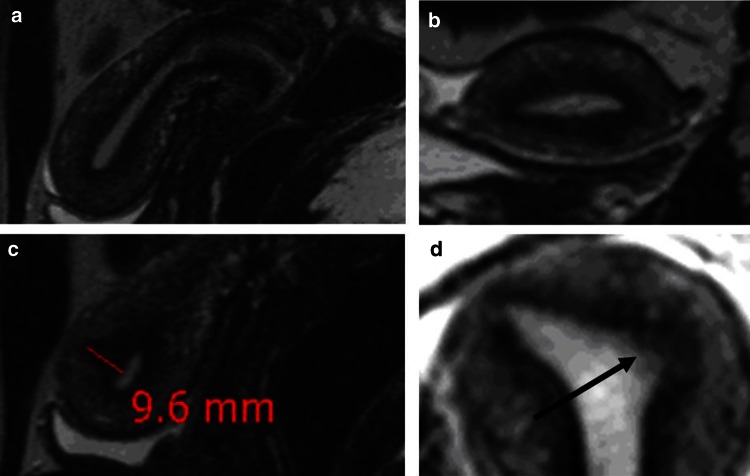
MRI scans of a 25 years old patient with extreme primary dysmenorrhea as an example for the establishment of the diagnosis of adenomyosis by ‘visualisation’. The diameter of the JZ in the mid-sagittal plane would not allow for the diagnosis of adenomyosis (**a**). A closer analysis of an enlargement of the JZ in the left cornual angle (**b** and **c**) revealed beginning cystic cornual angle adenomyosis (**d**) (arrow)

Adenomyosis constitutes an alteration of the archimetra that begins with the local invasion of basal endometrial glands and basal stroma into the underlying archimyometrium disrupting focally the continuity of this innermost muscular layer of the myometrium. In transvaginal sonography (TVS), this phenomenon may be detected as a focally destroyed ‘halo’ and an irregular endometrial–myometrial interface. In MRI a diameter of the junctional zone (JZ) of ≥12 is suggested to warrant an assured diagnosis of uterine adenomyosis. Adenomyotic lesions of that size certainly do no constitute beginning, but rather advanced lesions. In the context of our study, it was necessary to identify lesions that might present with a thickness of the JZ of 8–10 mm or even below. A representative example is shown in Fig. 3. While in the mid-sagittal plane no evidence of adenomyosis could be demonstrated, discrete signs of adenomyosis could be detected in the left cornual angle.

**Fig. 4:**
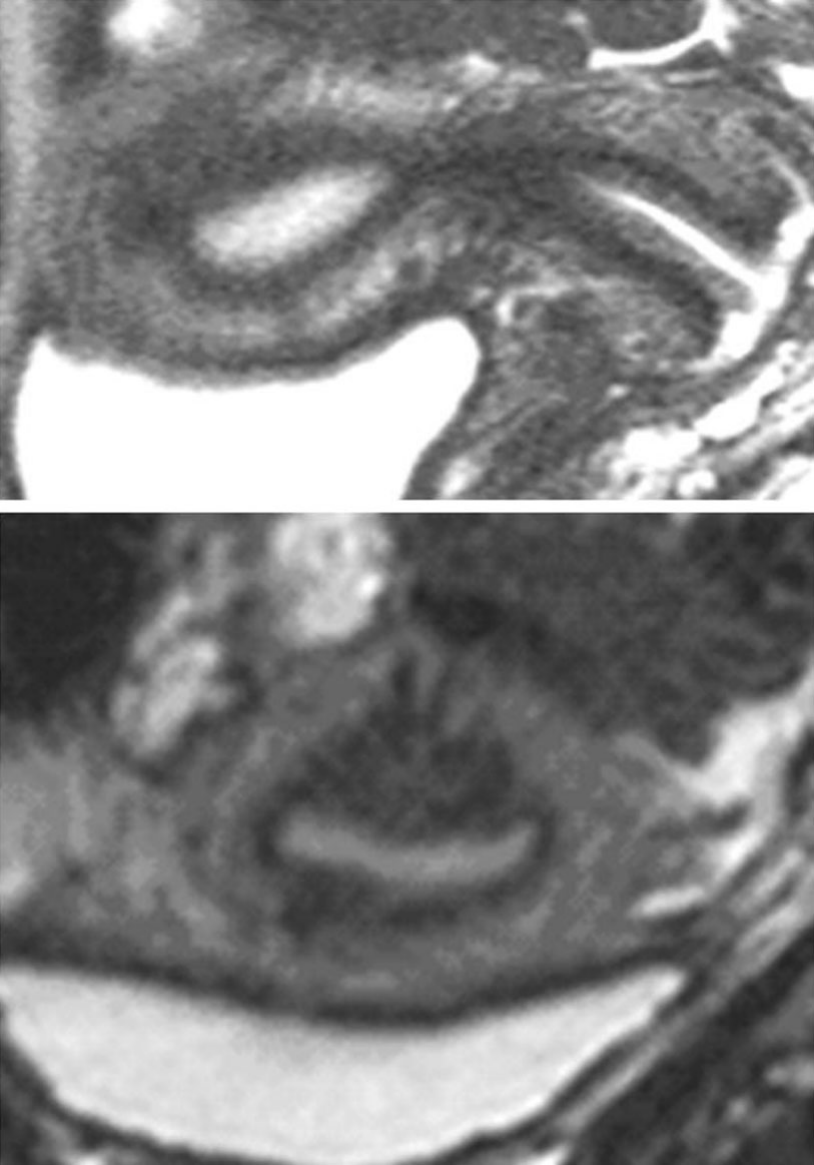
Adenomyosis in a 32-year-old woman without dysmenorrhea. In the sagittal scan the hypointense area could result from an episodic neometral contraction (*top*). The coronal scan performed after a short lapse of time, however, reveals adenomyosis with signs of spread of the lesion in various directions (*bottom*)

Despite spasmolytic effects of butylscopolamine, transient alterations of the JZ, such as peristaltic waves or episodic contractions of the neometra, may occur and have to be excluded. Persistence of such alterations after a lapse of time is indicative of adenomyosis (Fig. 4).

**Fig. 5:**
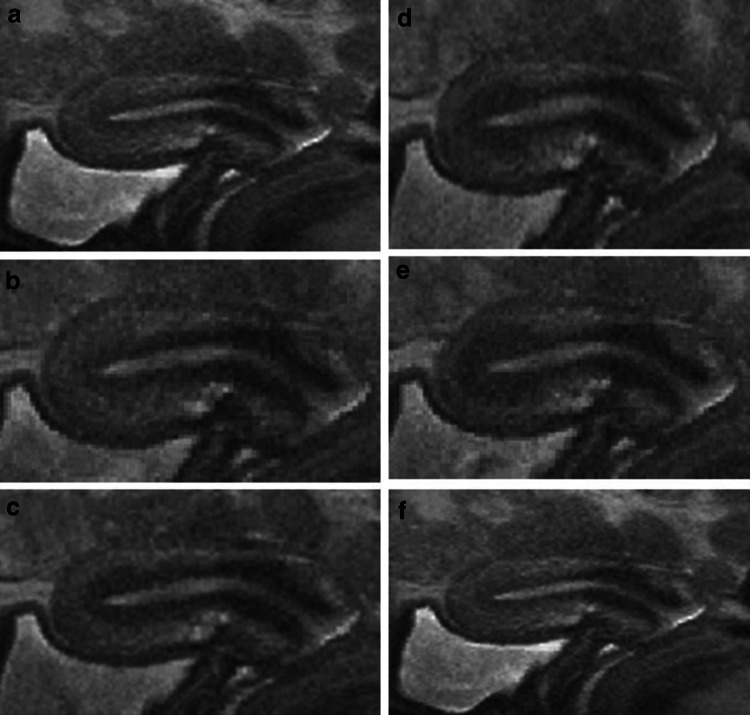
The course of a peristaltic wave of the archimyometrium as shown by a sequence of MRI scans obtained from cinematographic MRI scan in a healthy woman in the late follicular phase. Initially, the archimyometrium appears to be relaxed, indicated by a thin JZ with a less marked hypointensity (**a**). The peristaltic wave starts with tension of the archimyometrium in the lower half of the uterine corpus, indicated by marked hypointensity of the JZ (**b**). The zone of increased tension (marked hypointensity) moves in a fundal direction. A muscular package is built up, indicated by the rapid increase of the JZ as the wave moves in a fundal direction (**c**–**e**) followed by a rapid relaxation (**f**)

A peristaltic wave is initiated with a contraction of the archimyometrium on the level of the lower uterine segment indicated by a decrease in the intensity of the JZ in MRI. The JZ becomes symmetrically thicker as the peristaltic wave moves toward the fundal part of the uterus, involving also the fundal aspects of the cornua and leaving a relaxing archimyometrium behind. At the height of the wave, the JZ reaches a thickness of about 10–12 mm. Thereafter, the archimyometrium is completely relaxed, as indicated by an increase in the intensity of the whole JZ (Fig. 5).

### Prevalences of adenomyosis vs endometriosis

The prevalence of adenomyosis in endometriosis and vice versa was calculated on the basis of the diameter of the junctional zone being ≥8, ≥10 and ≥12 mm, respectively, in the mid-sagittal or paramedian planes. The prevalence of endometriosi in adenomyosis (*n* = 72) increased from 75.5 to 77 % and 78 %, respectively, and the prevalence of adenomyosis in endometriosis (*n* = 56) declined from 92.5 % to 79 % and 59 %, respectively, with the limits of diagnosis of adenomyosis being set at ≥8, ≥10 and ≥12 mm of junctional zone width, respectively (Table 3).

**Table 3:** The prevalences of endometriosis in adenomyosis and vice versa as determined on the basis of arbitrary detection limits with respect to the thickness of the junctional zone at ≥8, ≥10 and ≥12 mm, respectively

Prevalence of endometriosis in adenomyosis		
JZ thickness	*n*	%
≥8 mm	50/66	75.8
≥10 mm	44/57	77.2
≥12 mm	33/42	78.6

Prevalence of adenomyosis in endometriosis		
JZ thickness	*n*	%
≥8 mm	52/56	92.9
≥10 mm	44/56	78.6
≥12 mm	33/56	58.9

Prevalence of endometriosis in "visualized" adenomyosis and vice versa		
	*n*	%
Endometriosis in visualized adenomyosis	54/67	80.6
Visualized adenomyosis in endometriosis	51/56	91.1

In “visualized” adenomyosis, the diagnosis is based upon a thickness of the JZ of ≥12 mm and criteria characteristic of adenomyosis below this limit of detection

Since criteria suggestive of the diagnosis of adenomyosis do exist in addition to the diameters of the junctional zone of the anterior and/or posterior wall as demonstrated in sagittal and/or coronary planes, respectively, a meticulous analysis of the MRI scans was performed in all three planes, the sagittal, coronary and transverse one, respectively.

Using this method of ‘visualization’ as a diagnostic tool in addition of the junctional zone diameter 127 (89 %) of the 143 patients studied fulfilled the diagnostic criteria of presenting with adenomyosis. In this group of patients the presence or absence of endometriosis was documented in 67 patients. Fifty-four patients presented with and 13 without endometriosis. Thus, the prevalence of endometriosis (*n* = 54) in visualized adenomyosis (*n* = 67) was 80.6 %. In the 56 total cases of documented endometriosis, 51 patients presented with adenomyosis determined by the method of ‘visualization’. Thus, the prevalence of visualized adenomyosis in endometriosis was 91.1 % (Table 3).

### Location, shape and patterns of the adenomyotic lesions

To obtain a better understanding of the possible modes of auto-traumatization, the localizations, shapes and patterns of the individual adenomyotic lesions were analyzed by reviewing the sagittal, coronary and transverse planes, respectively, in each patient. The data are summarized in Table 4. Nearly all of the lesions were localized in the longitudinal midline of the uterus with extensions into the paramedian planes and to the fundal myometrium. Lateral extensions of the lesions into the cornual and lateral regions caudal to the cornua were in general expansions of lesions that originated in the mid- and paramedian plane. Isolated endometriotic cysts were found in 27.3 % of the cases. They were located in a more or less haphazardly scattered way within the expanded junctional zones of the anterior and posterior wall as well as of the fundal region.

**Table 4:** The median, paramedian, lateral and fundal localizations of the endometriotic lesions within the myometrium of the uterine corpus

Localization	*n*	%
Fundal	69	49.6
Median	130	93.5
Paramedian	96	69.1
Lateral	23	16.5
Cornual	34	24.5
Cornual cysts (total)	22	15.8
Cornual cysts (accumul.)	11	8.7
Other cysts	38	27.3

As indicated by the localization of the largest diameter of the JZ, the majority of the individual lesions originated in the mid-sagittal region of the anterior and/or posterior wall of the uterus. Also, the fundal and paramedian regions were major primary localizations. Adenomyotic lesions did not primarily develop at the lateral uterine wall or in the cornual region. Adenomyosis at these localizations constituted lateral or cornual extensions of large adenomyotic lesions that primarily originated within the anterior or posterior wall. Cystic structures were a frequent finding. Cysts that accumulated in one or both cornua, however, constituted a distinct entity. The findings exceed 100 % because individual adenomyotic lesions displayed more than only one of these criteria

**Fig. 6:**
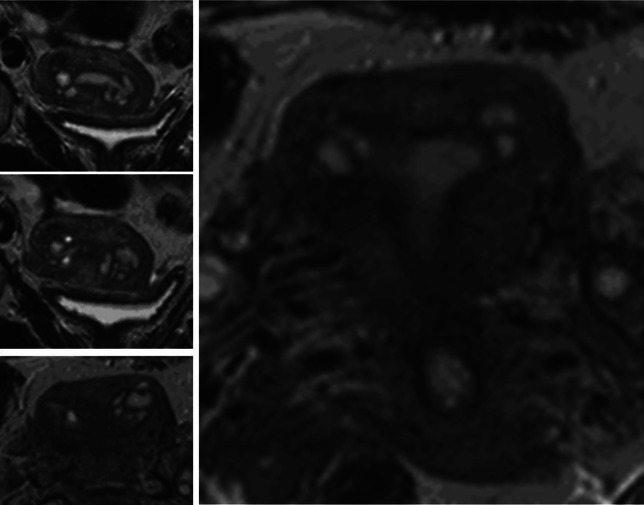
Cystic coronary angle adenomyosis in an infertile woman aged 40 years with extreme primary dysmenorrhea and documented endometriosis. In the sagittal midline of the uterus, no enlarged JZ could be demonstrated

**Fig. 7:**
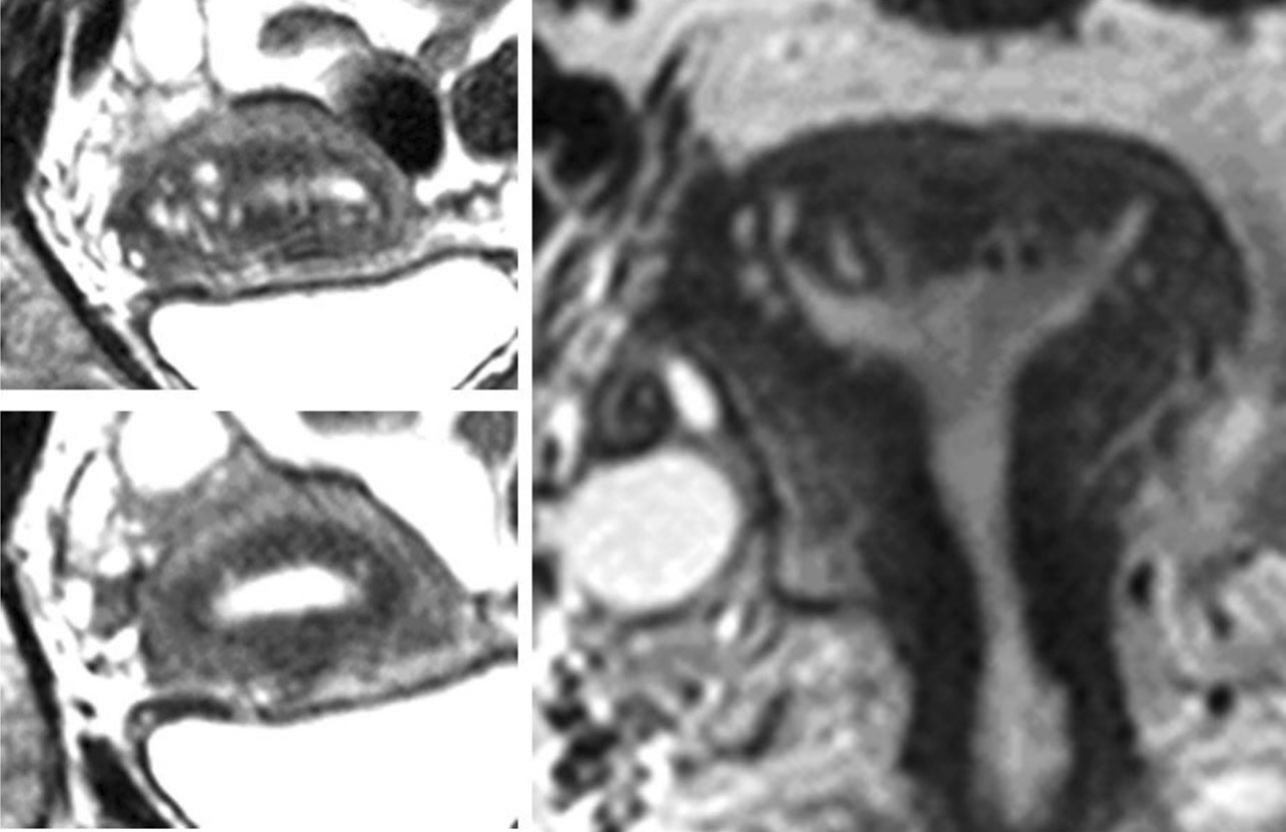
Cystic cornual angle adenomyosis in an infertile woman aged 39 years with extreme primary dysmenorrhea. Laparoscopy was not performed. In addition to the cornual cysts, there is an enlargement of the JZ in the midline of the anterior uterine wall (coronary plane; *bottom left*)

**Fig. 8:**
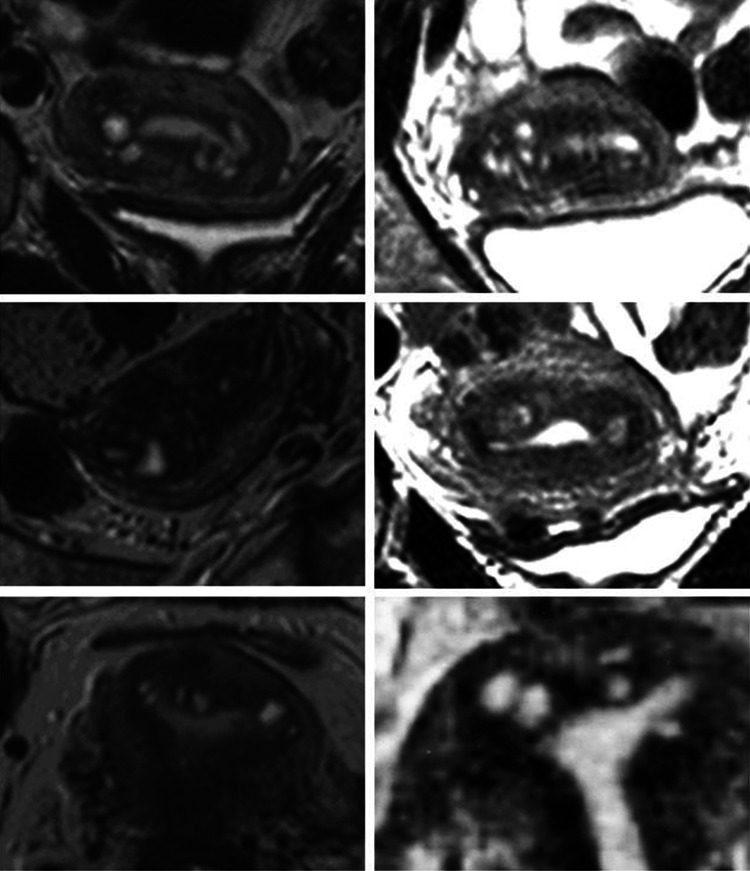
Six examples of cystic cornual angle adenomyosis. These women suffered from extreme primary dysmenorrhea

In 15.8 % (*n* = 22), cystic structures were identified in one or both cornua of the uterus. An accumulation of endometriotic cysts within adenomyotic lesions in the cornual region of the uterus was found in 11 cases and appeared to constitute a distinct phenomenon. They comprised about 8.7 % of the total cases of adenomyosis studied and were only observed in women with extreme primary dysmenorrhea (Table 4; Figs. 6, 7, 8).

**Fig. 9:**
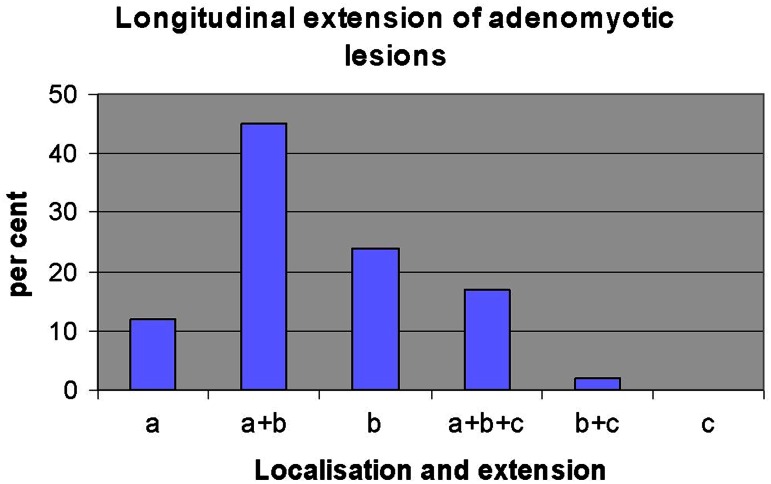
The longitudinal extension of adenomyotic lesions in a percent of the cases in the upper third (*a*), middle third (*b*) and lower third (*c*) of the uterus. Adenomyotic lesions were localized predominantly in the upper two-thirds of the uterine corpus and extended also over the whole length of the uterine corpus (*a*+*b*+*c*). They rarely presented in the lower two-thirds (*b*+*c*) and never in the lower third (*c*)

Also, the extension of adenomyotic lesions along the longitudinal axis of the uterus was considered to provide additional information with respect to localizations of mechanical strain imposed on the archimetra. Eighty-one percent of the adenomyotic lesions presented at the level of the upper and middle third of the uterine cavity. About 17 % extended over the whole length and only 2 % on the lower two-thirds of the uterine cavity. No adenomyotic lesion was observed that presented only on the level of the caudal third of the uterine cavity (Fig. 9).

## Discussion

The association of adenomyosis with endometriosis was studied in the past with extremely diverging results. In older reports [40] and in a large review, the prevalence of endometriosis in adenomyosis ranged from about 10 to 80 % [20]. The reported data were most probably based upon casual findings during surgery and not upon data collected in a specific study focusing on that issue. Most of the patients studied were in an advanced stage of their reproductive life; superficial endometriotic lesions might have disappeared at that time and smaller infiltrative lesions such as in the sacro-uterine ligaments might have been left unrecognized during surgery. Thus, most probably only the persistent and the more impressive lesions were noticed. The recognition of small endometriotic lesions had to await laparoscopy and a system of classification that takes a wide range of lesions, including minor ones, into consideration [23]. It is not surprising that in a systematic study focusing on the association of endometriosis with adenomyosis, the prevalence of uterine adenomyosis in endometriosis was exceptionally high [41].

With respect to MRI, at present, the number of studies concerning the association of adenomyosis with endometriosis is limited [5, 37]. The discrepant findings with regard to the prevalence of adenomyosis in endometriosis and vice versa may be due to the radiological criteria chosen that have to be fulfilled for the accurate diagnosis of adenomyosis [33–35]. Strict criteria are important in radiological routine diagnosis, as they help to prevent false-positive results that might lead to a non-indicated surgical intervention. Within the context of a scientific study dealing with the pathophysiology of adenomyosis and endometriosis, strict criteria may conceal the true nosological relationship between these lesions. It is obvious that uterine adenomyosis starts as a small lesion and may expand thereafter to larger focal or diffuse ones at various velocities. The same understanding of the development of adenomyosis might have prompted Bird and co-workers to include sub-basal adenomyotic lesions in their study on the prevalence of adenomyosis in sequential hysterectomies [42]. Sub-basal adenomyosis does not fulfill the usual morphological criteria required for the establishment of the diagnosis [43]. The significance, however, of sub-basal adenomyosis might be derived from the fact that, although the prevalence was only in the range of 30 %, these lesions were symptom productive in 70 % of the cases with irregular bleedings constituting the most prominent one [42].

In a previous MRI study that used the criterion of ≥12 mm of JZ thickness to establish the diagnosis of adenomyosis, the prevalence of adenomyosis in endometriosis was reported to be in the range of 34.6 % in comparison to healthy controls with a prevalence of 19.4 % [37]. In our study, with the same criterion used, the prevalence of adenomyosis in endometriosis is 58 %. In view of the fact that adenomyotic lesions develop from small ones and present with a diameter of the JZ at ≥12 mm only after considerable growth, it therefore has to be emphasized that the prevalence of adenomyosis in endometriosis is at least 58 %. In our previous and present study, with a width of the JZ of ≥10 mm as the lower limit for the diagnosis of adenomyosis, a prevalence of 79 % was found [5] supporting this argument. If “visual” adenomyosis is included, the prevalence of endometriosis in adenomyosis is 80.6 % and of adenomyosis in endometriosis is 91.1 %, respectively. (Table 3)

**Fig. 10:**
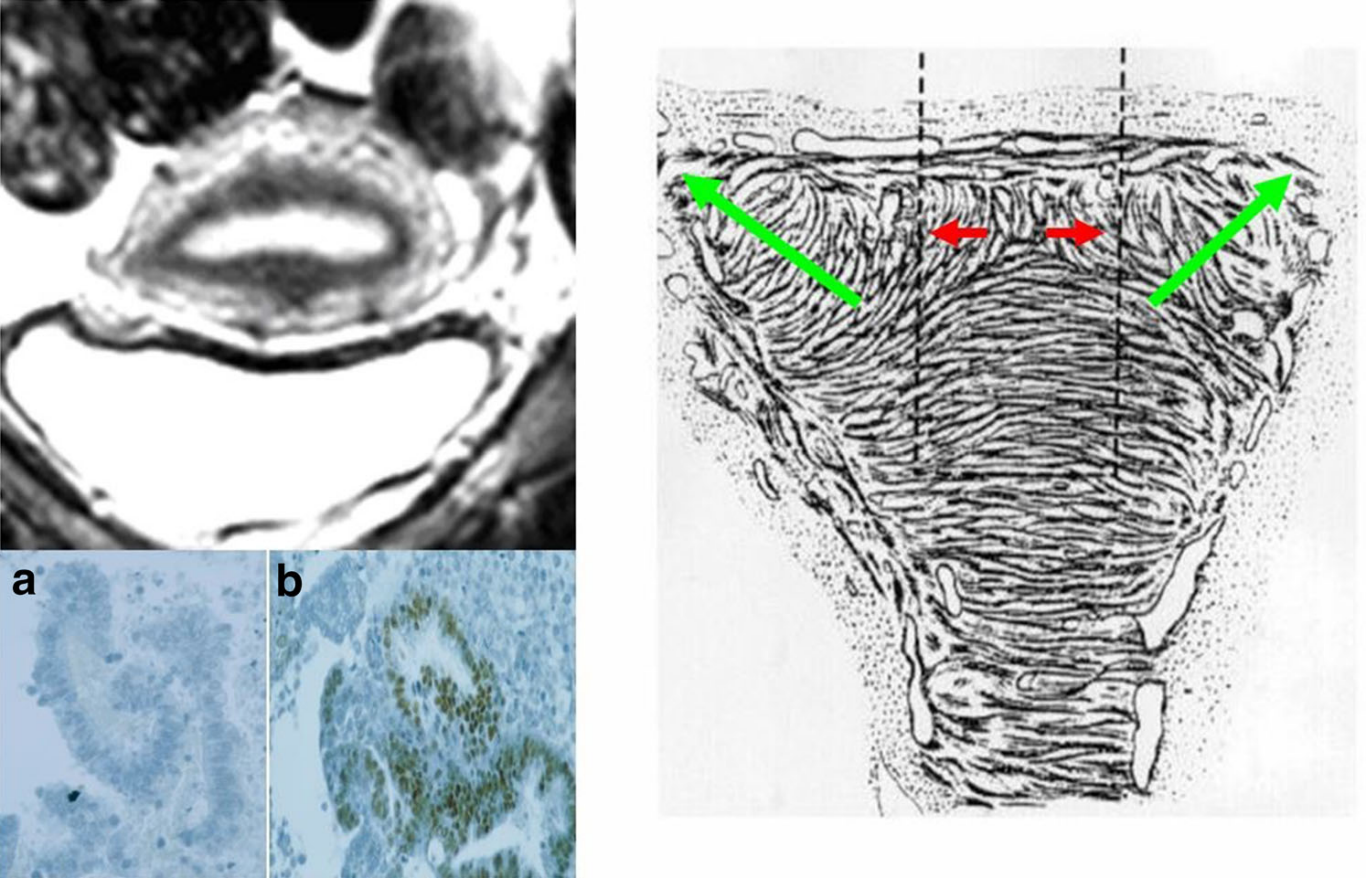
Schematic representation of the mechanism of uterine auto-traumatization by uterine peristalsis and hyperperistalsis at the fundo-cornual raphe. *Green arrows* direction of sperm transport. *Red arrows* distraction of basal stromal cells and archimyometrial myocytes at the fundo-cornual raphe by uterine peristalsis. With the development of an early adenomyotic lesion in the midline of the upper uterine corpus, a chronic process of proliferation and inflammation is established that facilitates the detachment of the basal endometrium. Fragment of detached functionalis (**a**) and a fragment of detached basalis (**b**) in menstrual blood (modified from [2, 4, 7])

We had suggested that uterine peristalsis and hyperperistalsis constitute the main mechanisms of uterine auto-traumatization [7, 8] (Fig. 10). On the basis of new and unusual findings of adenomyosis in MRI, such as a surprising accumulation of cornual endometrioma [9] and adenomyosis in uteri with malformations that lack a fundo-cornual raphe [44, 45], we had to extend this view and take another important mechanical function of the non-pregnant uterus into consideration that had escaped our attention before. With respect to the development of adenomyosis, this mechanism of uterine auto-traumatization was termed the ‘archimetral compression by neometral contraction’ [9]. To identify the specific impact of peristalsis/hyperperistalsis and neometral contraction within the TIAR mechanism these two functions had to be analyzed in detail and correlated with the localization, shape and pattern of the individual adenomyotic lesions.

With respect to uterine peristalsis, the structure of the stratum subvasculare (archimyometrium) that is involved in directed sperm transport had been described by Werth and Grusdew [46] and in our own previous publications [8, 36, 47]. The myocytes of the archimyometrium are densely packed with little interstitial tissue [48]. In cinematographic MRI, the peristaltic waves start near the internal os of the cervical canal and rapidly move in the fundal direction (Kissler, S., personal communication; unpublished video) (Fig. 5). Probably by the activation of both the circular and short longitudinal fibers [46], a muscular package is built up as the wave moves in the fundal direction providing the pressure and power that enable this peristaltic pump in the late follicular phase to inject sperm into the beginning of the ampullary part of the tube leading to the dominant follicle [29, 30]. Thus, this peristaltic pump may exert its strongest power at the upper level of the uterine cavity (Fig. 5).

If a mean frequency of 1.5 waves of peristaltic contractions per minute over the whole cycle is assumed [49], nearly 10 million peristaltic waves occur during the first 10 years after menarche and about 30 million during the whole reproductive period of life.

Astonishingly, neometral contractions at the end of ovulatory cycles have long not been considered as an important mechanical function of the non-pregnant uterus. During an ovulatory cycle, the oxytocin receptors (OTR) accumulate in the stratum vasculare of the myometrium. Their formation is stimulated by follicular estrogen and by the subsequent increase of luteal progesterone [50]. Following the decline of progesterone in blood, the OTRs are activated presumably by endometrial oxytocin (OT) [51]. A gradient of OTR concentration along the longitudinal uterine axis with highest density of the OTR in the fundal part of the uterus [52] ensures the orthograde discharge of menstrual debris and at the same time these contractions may occlude the intramural part of the tube, thus presumably minimizing the efflux of menstrual debris into the peritoneal cavity. The muscular mass of the neometra that is highest in the fundal region of the uterus [48, 53] and the increased concentration of OTRs strongly suggest that the power of the neometral contractions during menstruation and the impact on the archimetra are highest in the fundal region of the uterus. Primary dysmenorrhea of young women is caused by these contractions and is experienced usually over a period of 24–48 h [39]. Measurements of intrauterine pressure indicate that these contractions occur at a frequency of about four to five per 10 min [54, 55]. Assuming a mean duration of these contractions over a period of 36 h, about 1,000 single contractions occur at the end of each ovulatory cycle, more than 130,000 during the first 10 years after the onset of ovulatory cycles and about 0.5 million contractions during the reproductive period of life.

Thus, both of these mechanical functions of the non-pregnant uterus appear to impose an enormous chronic mechanical strain on the subendometrial stromal cells that may result in local inflammation and proliferation of basal endometrium into the uterine wall with the development of adenomyotic lesions and at the same time in dissemination of fragments of basal endometrium into the peritoneal cavity and at distant sites of the body [7, 8].

The majority of the adenomyotic lesions are localized on the level of the upper two-thirds of the uterine cavity (Fig. 9). This is in keeping with the assumption that both mechanical functions of the non-pregnant uterus exert their strongest power on the upper part of the uterus. Also, the predominant localization of adenomyotic lesions at or near the midline of the upper anterior and/or posterior walls of the uterus and particularly that of early lesions close to the fundo-cornual raphe correspond to the mechanism of both functions [6, 7] (Fig. 10).

Cystic cornual angle adenomyosis had already been observed by Friedrich von Recklinghausen. That he did not consider this variety just as one of many others, but rather as a specific form of adenomyosis may be derived from the fact that he devoted two special chapters of his book to this kind of lesion, which he coined “Zystische Tubenwinkeladenomyose” (cystic cornual angle adenomyosis) [11].

**Fig. 11:**
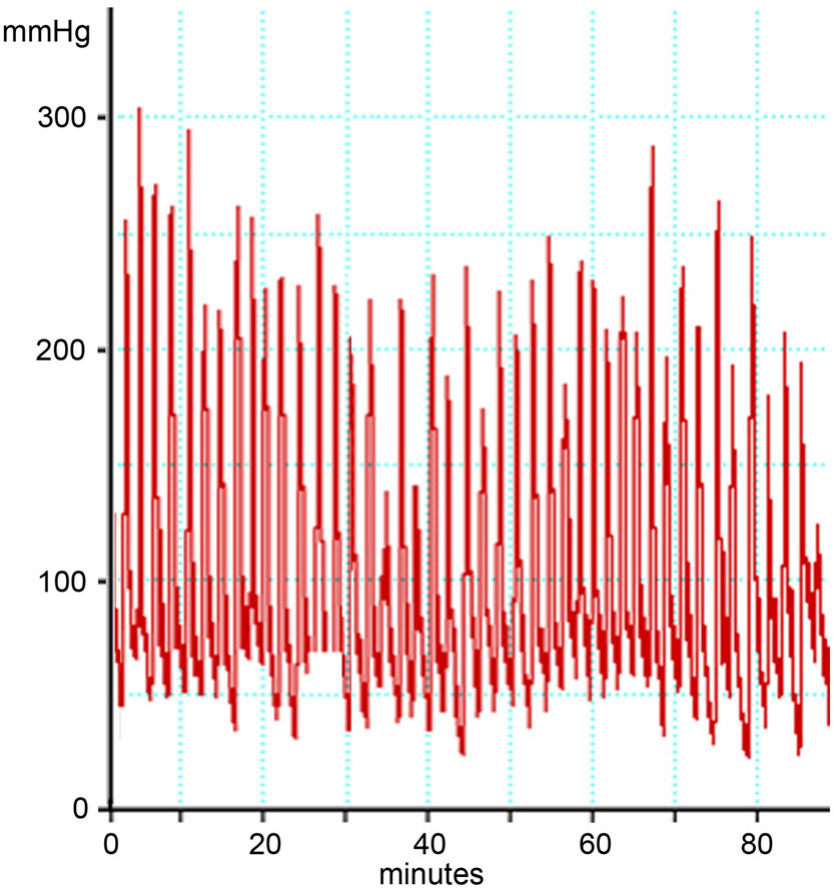
Recording of intrauterine pressure in an adolescent girl with extreme primary dysmenorrhea on the second day of the cycle (Courtesy L. Wildt and B. Böttcher)

In our study we observed 11 cases of adenomyosis that most probably correspond to the cases described by von Recklinghausen. These adenomyotic lesions were characterized by the occurrence of multiple cysts, either in the cornual region of one side or mostly in both cornua. In most of these cases, also an expansion of the JZ in the mid-sagittal plane was observed. The most striking finding was that these women were exclusively suffering from extreme primary dysmenorrhea. In these women the intrauterine pressure may reach 300 mmHg and may not fall below 100 mmHg between single contractions (Fig. 11). The extreme pain most certainly results from both, the strong pressure and also the concomitant ischemic insult.

**Fig. 12:**
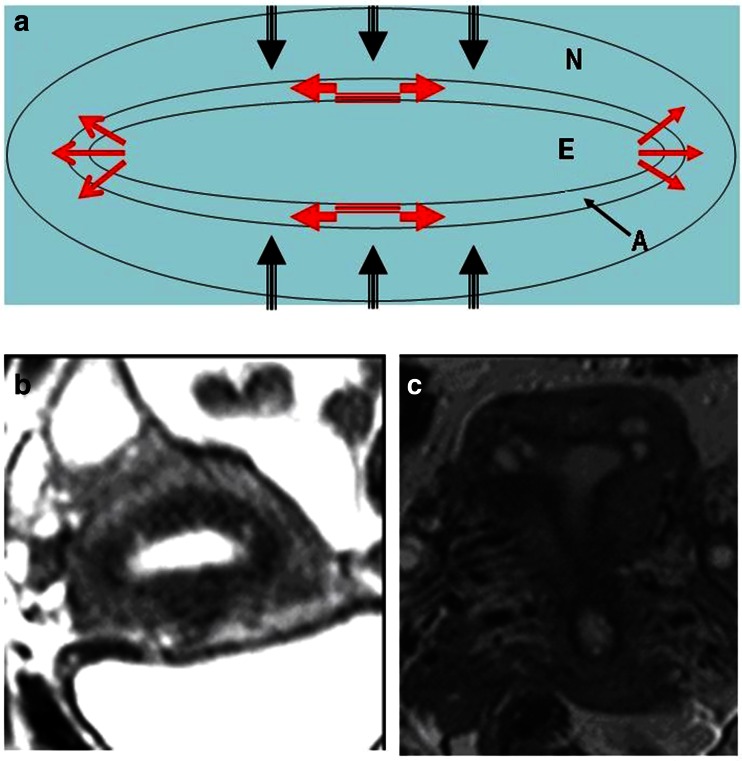
Schematic representation of uterine auto-traumatization by the mechanism of ‘archimetral compression due to neometral contraction’ at the onset of menstruation. *N* neometra; *E* endometrium; *A* archimyometrium (**a**). Due to the high intrauterine pressure in consequence of the contraction of the neometra, the archimyometrium ruptures in the cornual angles and fragments of basal endometrium are dislocated into the myometrial wall, where they develop into endometriotic cysts (**b** and **c**). At the same time, basal stromal cell at the fundo-cornual raphe are chronically overstretched resulting in the initiation of the TIAR mechanism and the development of an adenomyotic lesion

In Fig. 12 it is attempted to illustrate our view of the mechanism of uterine auto-traumatization by strong neometral contractions that severely compress the archimetra. In addition to the distraction of the stromal cells and myocytes of the archimyometrium in or close to the upper midline, the endometrium is pressed into the cornua of both sides. The archimyometrium in the cornual region is disrupted and fragments of basal endometrium are dislocated into or underneath the archimyometrium, where they grow and form intrauterine cystic endometrioma. That these cysts do not result from glandular infiltration into the myometrium, but rather from displacement of endometrial fragments may be derived from the observation that, in MRI, they are completely surrounded by their own junctional zone without any communication with the uterine cavity (Figs. 6, 7, 8). The astonishingly high association of cystic cornual adenomyosis with extreme dysmenorrhea not only supports the view that this form of adenomyosis is caused by the mechanism described above, but also strongly corroborates our pathophysiological concept of uterine auto-traumatization as the cause of the development of adenomyosis in general.

At present, one can only speculate which of these two mechanical functions of the non-pregnant uterus plays the predominant role in uterine auto-traumatization and the activation of the TIAR mechanism. With about 20–40 mmHg, even following the administration of oxytocin, the intrauterine pressure is comparably low in the late follicular phase of the menstrual cycle, when the peristaltic activity is highest [55]. Peristalsis and hyperperistalsis might exert their injuring effect on the archimetra by the distraction of sub-basal stromal cells and archimyometrial myocytes at the fundo-cornual raphe. In a previous report we had suggested that this might particularly pertain to anovulatory cycles, such as follicular persistency with high estradiol levels in blood and presumably long-lasting hyperperistalsis [7]. In any case, as suggested before, a longer phase of anovulatory cycles after menarche, before regular ovulatory cycles are established, might be considered as a critical phase with respect to the development of endometriosis and adenomyosis in young girls [56, 57].

Since 79 % of our patients were suffering from primary dysmenorrhea, we assume that “archimetral compression by menstrual neometral contractions” constitutes the predominant cause of uterine auto-traumatization with the subsequent formation of adenomyosis and endometriosis. Adenomyotic lesions observed in uterine malformations without a fundo-cornual raphe underline this view [44, 45]. Already, Freund described primary dysmenorrhea, in addition to bleeding disorders and infertility, as the main symptom of the patients he had studied together with von Recklinghausen [12]. In a recent report, a high proportion of young women with extensive endometriosis had a history of severe primary dysmenorrhea [58].

Additional mechanical strain may result from the disorganized muscular structure as soon as larger adenomyotic lesions are established. It had been earlier realized that uterine adenomyosis does not constitute a homogenous proliferation of endometrial glands with basal stroma and muscular tissue. If the latter would totally result from muscular metaplasia of the invading glandular stroma, one would expect a constant ratio between glandular and muscular tissue in adenomyotic lesions. This is not the case. While there are lesions that are completely pervaded by glandular and cystic structures, other lesions predominantly present with muscular tissue [13, 40]. It appears that in such cases the muscular proliferation is independent of the glandular one, resulting in irregularly arranged muscular fascicles that extend in various directions [59]. This may cause intralesional mechanical strain promoting the further expansion of the respective adenomyotic lesion. In real-time vaginal sonography and in cinematographic MRI (Kissler, S., personal communication, unpublished video), the irregular proliferation of the muscular component results in a convulsive pattern of the peristaltic activity and in a breakdown of directed sperm transport [30]. In summary, the non-pregnant uterus is constantly subjected to mechanical strain that bears the risk of auto-traumatization and the development of adenomyosis. As soon as adenomyotic lesions are in the process of development, further mechanical strain at various sites might arise that causes the pleiomorphic appearance of the individual adenomyotic lesions with proliferations in unpredictable directions (Fig. 4 from [5, 59]).

Sixty to seventy per cent of premenopausal women develop uterine adenomyosis [20, 42], which is, according to our findings, the consequence of the mechanical strain that is imposed upon the archimetra throughout the reproductive period of life. It has been claimed that premenopausal adenomyosis is not associated with endometriosis and that both lesions constitute different disease entities with no shares of a common pathophysiology [60, 61]. In studying whether or not a long period without childbirth is a risk factor for developing endometriosis, it could be shown that after a lapse of time of 10 and more years after the last childbirth, the prevalence of endometriosis was 26.8 % in symptom-free women at tubal sterilization. The mean age of the patients was 37.8 years [62, 63]. At this age a considerable number of women develop premenopausal uterine adenomyosis [64]. Although the endometriotic lesions in this study were predominantly described as minimal and mild, the results suggest that an association of endometriosis with adenomyosis and vice versa appears to be present throughout the whole reproductive period of life. In recent studies with hysterectomies performed because of uterine adenomyosis, a significant association of adenomyosis with endometriosis was reported [65, 66].

More extensive endometriotic lesions in association with uterine adenomyosis, however, appear to develop early during reproductive life [56–58]. Our previous and present data show that the early onset of the disease process is presumably caused by an increased mechanical strain that is imposed on the archimetra in consequence of intensified mechanical functions of the non-pregnant uterus with neometral contraction probably prevailing over archimyometrial peristalsis.

At this point of the discussion, it appears to be necessary to reflect on the possible etiology of the increased mechanical strain in young women that leads to early onset adenomyosis and endometriosis. Both mechanical functions of the non-pregnant uterus, uterine peristalsis and neometral contraction are under the control of oxytocin. Primary dysmenorrhea can be alleviated by the administration of oxytocin antagonists [54] and the frequency of uterine peristalsis can be increased by bolus injections of oxytocin [67]. Messenger RNA of oxytocin (OT) and its receptor (OTR) has been shown to be present in the endometrium of rodents [51]. Also in humans, OTRs have been shown to be expressed in the epithelium of the basalis layer [68]. OTRs are expressed in the neometra of the non-pregnant uterus [52] and also in the peristromal myocytes of endometriotic and adenomyotic lesions [69, 70]. Since the muscular tissue of these lesions is homologous to the archimyometrium [4], it may be assumed that they are also expressed in the archimyometrium of unaffected women. It had been calculated that OT produced by the endometrium by far exceeds the secretion of pituitary OT and it was therefore proposed, based on the data obtained in rodents, that the OT of endometrial origin and/or the products of an endometrial autocrine/intracrine OT/OTR interaction such as PGF2α control the contractile functions of the uterus [51]. In this respect, it is of interest to note that in women with endometriosis and adenomyosis, the layer of basal endometrium distant from adenomyotic lesions was double as thick as in women without the disease [4]. This could result in an increased production of OT in these women. Whether the enlarged basal endometrium and also the observation of an increased density of OTR in the myometrium of women with adenomyosis and severe primary dysmenorrhea [71] precede the onset of the disease and may constitute a genetic predisposition or constitute a sequel cannot be decided at present. A primarily hyperactivated uterine peristalsis on the basis of a primarily increased endometrial OT production could also provide a better understanding for the development of premenarchal endometriosis [72, 73] rather than a haphazard detachment and transtubal dissemination of basal endometrial fragments in these girls. In any case, the OT/OTR/PGF2α system could become a focus of research for the further elucidation of the molecular biology of early-onset adenomyosis and endometriosis.

The process of continuous proliferation and expansion of archimetral tissue in consequence of chronic mechanical strain and injury deserves some further attention. We assume that this phenomenon is due to the fact that, in the archimetra, HOX genes, such as HOXA 10, are constantly operative [74]: (1) the endometrium is cyclically regenerating after desquamation of the decidua; (2) there is cyclical metaplasia of stromal fibroblasts into archimetral myocytes and back into fibroblasts and (3) the process of decidualization is initiated at the end of the luteal phase to prepare the archimetra for implantation and, in case of failed conception, terminated following the decline of progesterone in blood and the desquamation of the functionalis layer. It is evident that these morphological and functional changes are controlled by ovarian steroids. Since HOXA 10 mRNA is up-regulated by estradiol [74, 75] it might be further up-regulated by the locally produced estradiol in consequence of the TIAR mechanism. The formation of endometriotic and adenomyotic lesions mimics the development of the respective eutopic structures during ontology. Already Cullen observed that some of the cystic and channel-like structures in adenomyosis constitute uteri “en miniature” [14]. With the formation of “mini primordial uteri” this phenomenon is also observed in peritoneal endometriosis [4]. Tissue injury and repair in adenomyosis, however, appears to have become excessive. With the formation of an adenomyotic lesion multiple sites of traumatization may emerge and at each of these sites processes of tissue injury and repair may be initiated anew, resulting in unexpected proliferations of the lesion in various directions [59] (Fig. 4).

## Conclusions

The present study confirms our concept that adenomyosis and endometriosis result from uterine auto-traumatization by physiological mechanical functions, such as uterine peristalsis for directed sperm transport and neometral contraction for the orthograde expulsion of menstrual debris at the end of the cycle. These functions may be exaggerated in women who acquire the disease early in their reproductive period of life. These findings not only provide new insights into the pathophysiology of adenomyosis and endometriosis, but also open up additional new avenues for the prevention and therapy of this disorder. Furthermore, with respect to the diagnosis of uterine adenomyosis (and consequently endometriosis), this study demonstrates a high degree of accordance between the results obtained in real-time TVS and MRI that might be further enhanced by clinical symptoms and historical data such as dysmenorrhea and infertility. Thus, the results of this study should encourage performing transvaginal sonography with care, to promote and advance early diagnosis of the disease by the general gynecologist.
